# Epidemiological and histopathological characteristics of head and neck cancers in Bhutan from 2011 to 2017: a retrospective descriptive study

**DOI:** 10.3332/ecancer.2020.1024

**Published:** 2020-04-15

**Authors:** Phub Tshering, Sithar Dorjee, Tshering Dendup, Thinley Dorji, Dechen Wangmo

**Affiliations:** 1 Jigme Dorji Wangchuk National Referral Hospital, Thimphu, Bhutan; 2 Khesar Gyalpo University of Medical Sciences, Thimphu, Bhutan; 3 Policy and Planning Division, Ministry of Health, Bhutan; 4 His Majesty’s Kidu Medical Unit, Thimphu, Bhutan; 5 Minister of Health, Royal Government of Bhutan, Thimphu, Bhutan

**Keywords:** head and neck cancer, Bhutan, epidemiology, histopathology, alcohol, tobacco, betel nut, oral cancer, thyroid cancer, laryngeal cancer, hypopharyngeal cancer, salivary gland neoplasm, nasopharyngeal cancer, sinonasal malignancy

## Abstract

**Background:**

Head and neck cancers are among the commonest cancers in the developing world. Personal habits, such as the use of tobacco, betel nut and alcohol are strongly associated with the development of head and neck cancers at certain sites. Therefore, they are among the preventable cancers. In Bhutan, there has not yet been a study conducted on head and neck cancers.

**Objective:**

To describe baseline epidemiological and histopathological characteristics of head and neck cancers in Bhutan.

**Methods:**

This is a 7-year descriptive study of all cases of head and neck cancers presented at the Jigme Dorji Wangchuk National Referral Hospital from 2011 to 2017. The data were collected from the hospital’s medical records section, histopathology records, patient referral unit and some treatment centres in India. Prior approval was sought from the Research and Ethics Board for Health, the Ministry of Health and the hospital management.

**Results:**

There were a total of 515 cases of head and neck cancers from 2011 to 2017. The crude incidence rate was 10 per 100,000 and the overall age adjusted rate was 12.3 (95% CI 9.5–15.1) per 100,000 population. The prevalence during this 7-year period was 69.1 per 100,000 population. The commonest cancers are thyroid, oral cavity, hypopharyngeal, laryngeal and nasopharyngeal cancer in decreasing order. Head and neck cancers are more common in males than females in the majority of sites except thyroid, salivary gland and sinonasal malignancies. Thyroid cancers and nasopharyngeal cancers are found to affect younger age groups. Tashigang (48) followed by Paro (43) recorded the highest number of cases. Squamous cell carcinoma is the commonest histopathology type in almost all the cases, while papillary carcinoma is the commonest among thyroid cancers. Personal habits, such as smoking, chewing tobacco, betel nut and alcohol consumption, were found to be more common among patients suffering from oral cavity, laryngeal, hypopharyngeal and oropharyngeal cancers.

**Conclusion:**

Head and neck cancers are the third most common cancer in Bhutan after stomach cancer and cervical cancer. Thyroid, oral cavity and hypopharynx are the top three anatomical sites for head and neck cancers in Bhutan. The current epidemiological and histopathological profile of head and neck cancers will form a baseline of information and basis for further research on head and neck cancers in Bhutan.

## Introduction

Bhutan is a small Himalayan country with an area of 38,394 sq Km and a total population of about 735,553 (National Statistical Bureau 2018). It has 20 districts and the health service is provided totally free through its national referral hospital, 2 regional referral hospitals, 30 district hospitals and 210 Basic health units. The national referral hospital in the capital is the only facility equipped for treatment of cancer and therefore gets referrals from across the country.

Head and neck cancers consist of a heterogeneous groups of cancers affecting different anatomical sites; oral cavity, nasopharynx, oropharynx, larynx, hypopharynx, nose and paranasal sinuses, major and minor salivary glands, thyroid gland and metastatic neck nodes of unknown primary. There are wide variations in the distribution of different types of head and neck cancers depending on their sociodemographic factors and lifestyle related risk factors [1]. The common risk factors for head and neck cancers are chewing of tobacco, smoking and alcohol [2–4]. Chewing of betel nut is also a significant risk factor for oral cavity cancer [5–7]. The carcinogenicity of these risk factors is dose dependent [3, 8]. Other risk factors are infection with HPV virus for oropharyngeal cancer and EBV for nasopharyngeal cancer [9–11].

Head and neck cancer is the third most common malignancy seen in both the sexes across the globe. ASR in Indian males exceed 30 per 100,000 and in Indian females exceed 10/100,000 [12]. The highest ASR of 63.58 in males was reported in Bas-Rin, France and the highest ASR of 15.97 in females in Madras, India [13]. Head and neck cancers are a significant problem in India constituting approximately one third of all cancer cases in contrast to 4%–5% in the developed world [14].

In Bhutan, The Population-based Cancer Registry (PBCR) started recording cancer cases in 2014 with support through the national Cancer Institute, Thailand and the International Agency for Research on Cancer. PBCR being relatively new, little effort has been made to analyse the data and no related scientific articles on head and neck cancer have been published. Until very recently, all the head and neck cancer cases were being referred to India at different centres and there is no access to data on any aspects of head neck cancers in Bhutan.

Therefore, this study aimed to establish a baseline epidemiological and histopathological profile of head and neck cancer patients in Bhutan. This study will provide information to guide further research, develop policy, help to implement strategic interventions for prevention and treatment, and create targeted awareness programmes in Bhutan.

## Materials and methods

### Study design

This was a retrospective descriptive study of all head and neck cancers for the last seven years between 2011 and 2017.

### Setting

The study was conducted at JDNWRH (National Referral Hospital), Thimphu. It is the only centre in the country for treatment of all types of cancers, and therefore all patients are referred to it from across the country.

### Study population and variables

All patients with a histopathological diagnosis of head and neck cancers were included in the study. Data were retrieved from the patient medical records, histopathological records, patient referral unit records and from treatment centres in India. Attempts were made to trace each of these 515 patients included in the study to fill in the missing information either by calling or following them in the Outpatient

Department. Variables collected for the study include patient name, age, sex, district, date of diagnosis, anatomical site of head and neck cancers, cancer types based on histopathological findings and risk factors, such as history of smoking, tobacco chewing, tobacco sniffing, betel nut chewing and alcohol intake).

### Data processing and analysis

Data were single entered into Epi Info Version 7.2.2. and analysis was done using STATA software version 13.0 (Stata Corporation, College Station, TX, USA). The ASR of head and neck cancer was estimated based on number of cases divided by population size based on the national census record (NSB, 2018). Descriptive statistics, such as frequencies of head and neck cancers, were presented based on year of diagnosis and districts of Bhutan, the anatomical sites by gender and age group, occupation, ethnicity, education level and histopathological classification. In addition, frequency of risk factors by cancer type based on anatomical sites was also described.

### Ethics approval

Ethical clearance was sought from the Research and Ethical Board for Health and administrative clearance from the national referral hospital.

## Results

**Figure 1.**
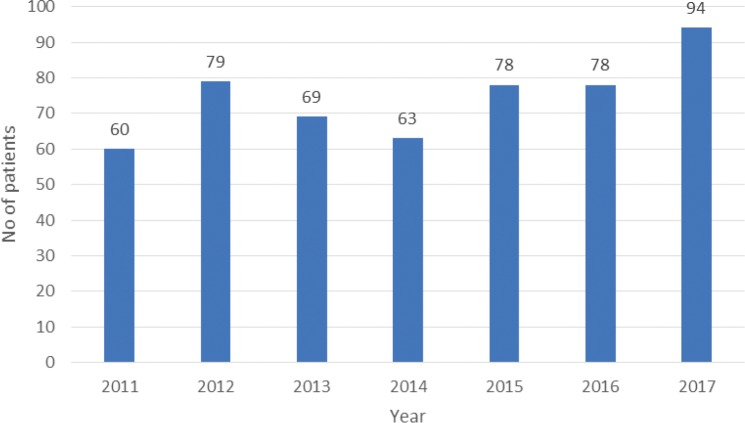
Trends in the number of head and neck cancers at the National Referral Hospital, Thimpu, Bhutan (2011–2017).

A total of 515 patients were diagnosed with a head and neck cancer from 1 January 2011 to 31 December 2017. The crude incidence rate was 10 per 100,000 and the overall age adjusted rate was 12.3 (95% CI 9.5–15.1) per 100,000 population. The prevalence of head and neck cancers is 69.1 per 100,000 population (Table 1). The yearly trend in the number of cases of head and neck seems over the study period indicated similar (Figure 1).

**Figure 2.**
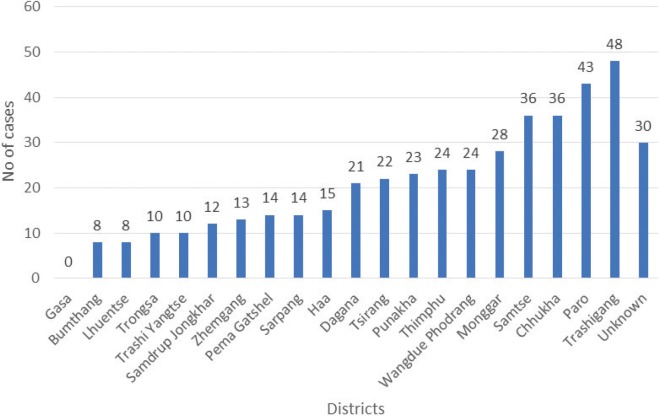
District wise distribution of head and neck cancers at the National Referral Hospital, Thimpu, Bhutan (2011–2017).

There were 263 males and 252 females with a mean age of 52.47 years (95% CI 51.03–53.9). About 42.8% of the patients are in the age group of 51 to 70 years (Table 2). Most of the patients are farmers (59.7%) and about 72.3% did not attend any schooling. Ethnically head and neck cancers are highest among the Sharchops compared to other ethnic groups (Figure 2).

The top five sites for head and neck cancers during the study period were thyroid (29%), oral cavity (20%), hypopharynx (12%), larynx (10%) and nasopharynx (9%). The commonest subsites for different sites are illustrated in Table 3. Sqamous cell carcinoma is the commonest histopathology type for oral cavity, hypopharynx, larynx, oropharynx, ear and unknown primary site accounting for 88.4% of the cases. For salivary gland cancer, the mucoepidermoid cancer is the commonest (43.7%), while papillary carcinoma thyroid is the commonest (82.6%) among the thyroid cancers. Other most rare histopathology types are illustrated in Table 4.

Among the risk factors, chewing of tobacco and betel nut was found to be more common among the oral cavity cancer cases. Smoking and consumption of alcohol are more common in hypopharyngeal, laryngeal and oropharyngeal cancers (Table 5).

## Discussion

This study describes the first baseline epidemiological and histopathological characteristics of head and neck cancers in Bhutan with a crude incidence rate of 10, ASR of 12.3 (95% CI 9.5–15.1) and prevalence of 69.1 per 100,000 population.

Head and neck cancers were observed more among males than females for all anatomical sites except for thyroid, major salivary glands and sinonasal malignancies. The males are three times more prone than females for laryngeal, hypopharyngeal and oropharyngeal. This is similar to other studies as reported by Bhattacharjee et al. [17]; Shunyu and Syiemlieh [6]; Tangjaturonrasme *et al* [15]; Delagranda *et al* [18]; Taziki *et al* [16]. This could be attributed mainly to the abuse of risk factors, such as tobacco, betel nut and alcohol consumption, where these risk factors were two times more prevalent in males than in females. On the other hand, thyroid cancers and salivary gland cancers are more common in females which could only be explained in terms of genetic predisposition.

The age range for development of head and neck cancers in general is between 7 and 89 years with maximum number of cases in between 40 and 65 years of age. Addala *et al* [19] and Krishna *et al* [20] found in their studies similar peak incidence between 40 and 65 years of age. For thyroid and nasopharyngeal cancers, it occurs even in the younger age group of 19 to 41 years as reported by Jung *et al* [21] in Korea.

The top three sites for development of head and neck cancer are thyroid, oral cavity and hypopharynx. In Thailand, Tangjaturonrasme *et al*. [15] found cancer of oral cavity, nasopharynx and larynx to be the commonest in males, while it is thyroid and oral cancers for females. Similarly, the commonest sites are oropharyngeal cancer in Northeast India [22], laryngeal cancer in London [23], nasopharyngeal cancer in Nigeria [24] and skin cancer of the head and neck region in Iran [25]. These variations can be explained by the differences in exposure to different risk factors. This is true even among the different subsites within the oral cavity. For instance , in studies by Leite *et al* [26] and Arora *et al* [7] , tongue is the commonest subsite, while Buccal mucosa is the commonest subsite in other studies [12, 19, 20].

One of the main reasons for increased thyroid cancer could be because of increase in the number of detection since more people seek medical attention for thyroid swelling. In Korea, where thyroid cancer is the commonest cancer, an improvement in diagnostic sensitivity and screening rates accounted for the increase in thyroid cancer rather than a true increase in its occurrence [21]. The abuse of habits, viz, smoking, tobacco chewing, betel nut chewing and alcohol consumption could be the main reason for other commoner cancers of head and neck, such as the oral cavity, hypopharynx, larynx and oropharynx. Such an association of these risk factors with head neck cancer was found in all previous studies [2, 3, 4, 27, 28]. A low BMI [9], Diets lacking fruits and vegetables and poor oral hygiene [26] and decreased socioeconomic status [10] were speculated to be some other risk factors for head and neck cancers though it couldn’t be probed in our study. In some cases, there are no known risk factors and such cases varies between studies with as low as 5.7% [20] to almost 60% of the patients with no known risk factors [29].

From among the districts, Tashigang has the highest case of head and neck cancer followed by Paro and Samtse. Compared to these eastern, western and southern districts, respectively, central districts, like Bumthang and Trongsa has comparatively fewer number of cases. Gasa the least populated district has so far no recorded cases of head and neck cancer although it may also be possible that some cases with unknown districts could be from Gasa. Interestingly, almost all cases of NPC are either from the southern or the eastern districts. There could be a play of genetic predisposition and differences in exposure to risk factors to account for this variation. The susceptibility to risk factors varies between races as shown in the differences between whites and blacks when exposed to the same risk factors, viz, smoking and alcohol [30].

Squamous cell carcinoma is the commonest histopathology. This is mainly because of squamous epithelial lining of the upper aerodigestive tract from which most of these head and neck cancers arise. This was true in all of the previous studies [16, 17, 18, 24]. For major salivary gland, the commonest histopathology is mucoepidermoid carcinoma, while for thyroid cancer the commonest is papillary thyroid cancer. Similar findings are reported by these studies [15, 31].

The study has captured country wide data of all the head and neck cancers as Jigme Dorji Wangchuk National Referral Hospital is the only hospital in the country where diagnosis and treatment of cancer all cancers are carried out. Therefore, it is inclusive of all head and neck cancers in Bhutan except for those few cases that may not have reported to any health centres or remained undetected. Hence, the estimates of incidence of head and neck cancers presented in the study are fairly accurate. The main limitation of the study is its retrospective nature thereby leading to missing information, especially those patients who have already expired and whose family members could not be traced even through phone calls. Another limitation is about the risk factors whose information was taken only for those cancer sites with proven association with the risk factors like tobacco. Therefore, we couldn’t get a control arm to see the association of these risk factors.

## Conclusion

The study provides baseline information on the epidemiological and histopathological characteristics of all the head and neck cancers in Bhutan. Head and neck cancers are the third most common cancer in Bhutan, with thyroid, oral cavity and hypopharynx being the top three cancers. This information will help us to design future head and neck cancer related studies in Bhutan. Additionally, the information from this study can be used to provide input for developing the national cancer control programme and develop strategies to prevent some of these preventable cancers through commencement of screening programmes.

## Conflicts of interest

None declared.

## Figures and Tables

**Table 1.** Average annual age adjusted head and neck cancers per 100,000 population of Bhutan based on Bhutan Census Data of 2017 and WHO Average Population size.

Age group	WHO Standard Population	Head and neck cancers (n)	Bhutan’s Population	Age-specific	Age-standardised
<15	26.11	0.285714	189417	0.2	3.9
15–19	8.47	1	68286	1.5	12.4
20–24	8.22	2	75415	3.0	24.9
25–29	7.93	5	79280	5.9	47.2
30–34	7.61	5	65180	7.5	56.7
35–39	7.15	5	55549	9.5	68.0
40–44	6.59	4	41495	8.6	56.7
45–49	6.04	6	35533	18.1	109.3
50–54	5.37	9	29317	29.2	157.0
55–59	4.55	8	23898	32.9	149.6
60–64	3.72	8	20711	38.6	143.7
65–69	2.96	7	14654	50.7	150.1
70–74	2.21	8	11468	67.3	148.7
75–79	1.52	4	7871	56.3	85.5
80–84	0.91	1	5397	18.5	16.9
85+	0.64	0	3674	3.9	2.5
Total	100.00	73	727,145	351.5	1,229.1

Crude incidence rate of head and neck cancer is 73/727145 * 100,000= 10.0 per 100,000 population. The overall age-adjusted annual incidence rates of head and neck cancer is 12.3 (95% CI 9.5–15.1) per 100,000 population.

**Table 2.** Age group distribution of head and neck cancers at the Jigme Dorji Wangchuk National Referral Hospital, Thimphu Bhutan, from 2011 to 2017.

Cancer site	Age groups				Total (n)
	7–18 years	>=19 years	>=41 years	>=65 years	
Oral cavity	0	12	50	40	102
Oropharynx	0	5	22	18	45
Nasopharynx	3	16	21	6	46
Hypopharynx	0	0	39	22	50
Salivary gland	0	5	10	12	27
Thyroid	2	89	46	13	150
Ear	0	0	2	4	6
Unknown primary	0	2	7	3	12
Total	6	131	233	145	515

**Table 3.** Distribution of all head and neck cancers in Bhutan from 2011 to 2017 at the Jigme Dorji Wangchuk National Referral Hospital by its site and subsite.

Head and neck cancer sites and subsites	Patient’s Sex		Total
	Male	Female	
Cancer of the oral cavity			
Anterior tongue	8	12	20
Buccal mucosa	19	14	33
Floor of mouth	2	2	4
Alveolus	16	4	20
Retromolar trigone	2	1	3
Hard palate	4	3	7
Lip	4	4	8
Upper GB sulcus	2	0	2
Subsite unknown	5	0	5
Total	62	40	102
Cancer of the oropharynx:			
Base of tongue	10	5	15
Tonsil	15	2	17
Posterior pharyngeal	3	0	3
Subsite unknown	5	4	9
Total	33	11	44
Cancer of the larynx			
Supraglottis	24	7	31
Glottis	5	3	8
Subglottis	2	0	2
Subsite unknown	8	1	9
Total	39	11	50
Cancer of the hypopharynx			
Pyriform sinus	40	11	51
Post cricoid	1	0	1
Posterior pharyngeal	0	1	1
Subsite unknown	6	2	8
Total	47	14	61
Cancer of major salivary glands			
Parotid	2	12	14
Submandibular	1	1	2
Subsite unknown	0	1	1
Total	3	14	17
Cancer of nasopharynx	31	15	46
Cancer of nose and sinuses	12	16	28
Cancer of the thyroid	23	127	150
Cancer of ear	5	1	6
Cancer of unknown primary	7	5	12

**Table 4.** Histopathological distribution among various site of head and neck cancers at the Jigme Dorji Wangchuk National Referral Hospital, Thimphu Bhutan from 2011 to 2017.

Histopathology	Cancer sites (*n*)									
	ORC	ORO	LAR	HYP	SG	SN	NP	Ear	Thyroid	CUP
Squamous cell carcinoma	**95**	**39**	**49**	**61**	0	**9**	0	**5**	0	**10**
Adenocarcinoma	0	0	0	0	0	2	0	0	0	0
Mucoepidermoid carcinoma	0	0	0	0	**7**	0	0	0	0	0
Adenoid cystic carcinoma	0	0	0	0	2	0	0	0	0	0
Verrucous carcinoma	5	0	0	0	0	0	0	0	0	0
Squamous cell carcinoma in situ	1	0	0	0	0	0	0	0	0	0
Spindle cell carcinoma	0	1	1	0	0	0	0	0	0	0
Acinic cell carcinoma	0	0	0	0	2	0	0	0	0	0
Carcinoma ex-pleomorphic	0	0	0	0	2	0	0	0	0	0
Oncocytic carcinoma	0	0	0	0	1	0	0	0	0	0
Melanoma	1	0	0	0	0	3	0	0	0	1
Nasopharyngeal carcinoma	0	0	0	0	0	0	**46**	0	0	0
Neuroendocrine carcinoma	0	0	0	0	0	0	0	0	0	1
Lymphoma	0	5	0	0	0	3	0	1	0	0
Lymphoepithelial carcinoma	0	0	0	0	0	1	0	0	0	0
Alveolar rhadomyosarcoma	0	0	0	0	0	1	0	0	0	0
Papillary carcinoma	0	0	0	0	0	0	0	0	**124**	0
Follicular carcinoma	0	0	0	0	0	0	0	0	13	0
Medullary carcinoma	0	0	0	0	0	0	0	0	6	0
Anaplastic carcinoma	0	0	0	0	0	0	0	0	7	0
Unknown	0	0	0	0	2	8	0	0	0	0
Total	102	45	50	61	16	27	46	6	150	12

ORC: Oral cavity, ORO: Oropharynx, LAR: Larynx, HYP: Hypopharynx, SG: Major Salivary glands, SN: Sinonasal, NP: Nasopharynx, CUP: Carcinoma of unknown Primary.

**Table 5.** Different Risk factors for the following head and neck cancer sites at the Jigme Dorji Wangchuk National Referral Hospital, Thimphu Bhutan from 2011 to 2017.

Risk Factors					
**Cancer sites**	**Smoking tobacco (*n*)**	**Chewing tobacco (*n*)**	**Sniffing tobacco (*n*)**	**Chewing betel nut (*n*)**	**Drinking alcohol (*n*)**
Oral cavity	17	28	4	41	26
Oropharynx	15	11	1	17	12
Larynx	14	6	1	16	17
Hypopharynx	18	14	1	25	20
